# The Human Innate Immune Protein Calprotectin Elicits a Multimetal Starvation Response in Pseudomonas aeruginosa

**DOI:** 10.1128/Spectrum.00519-21

**Published:** 2021-09-22

**Authors:** Cassandra E. Nelson, Weiliang Huang, Emily M. Zygiel, Elizabeth M. Nolan, Maureen A. Kane, Amanda G. Oglesby

**Affiliations:** a Department of Pharmaceutical Sciences, School of Pharmacy, University of Maryland, Baltimore, Baltimore, Maryland, USA; b Department of Chemistry, Massachusetts Institute of Technology, Cambridge, Massachusetts, USA; c Department of Microbiology and Immunology, School of Medicine, University of Maryland, Baltimore, Baltimore, Maryland, USA; University of Manitoba

**Keywords:** *Pseudomonas aeruginosa*, calprotectin, iron, polymyxin B, proteases, zinc

## Abstract

To combat infections, the mammalian host limits availability of essential transition metals such as iron (Fe), zinc (Zn), and manganese (Mn) in a strategy termed “nutritional immunity.” The innate immune protein calprotectin (CP) contributes to nutritional immunity by sequestering these metals to exert antimicrobial activity against a broad range of microbial pathogens. One such pathogen is Pseudomonas aeruginosa, which causes opportunistic infections in vulnerable populations, including individuals with cystic fibrosis. CP was previously shown to withhold Fe(II) and Zn(II) from P. aeruginosa and induce Fe and Zn starvation responses in this pathogen. In this work, we performed quantitative, label-free proteomics to further elucidate how CP impacts metal homeostasis pathways in P. aeruginosa. We report that CP induces an incomplete Fe starvation response, as many Fe-containing proteins that are repressed by Fe limitation are not affected by CP treatment. The Zn starvation response elicited by CP seems to be more complete than the Fe starvation response and includes increases in Zn transporters and Zn-independent proteins. CP also induces the expression of membrane-modifying proteins, and metal depletion studies indicate this response results from the sequestration of multiple metals. Moreover, the increased expression of membrane-modifying enzymes upon CP treatment correlates with increased tolerance to polymyxin B. Thus, the response of P. aeruginosa to CP treatment includes both single- and multimetal starvation responses and includes many factors related to virulence potential, broadening our understanding of this pathogen’s interaction with the host.

**IMPORTANCE** Transition metal nutrients are critical for growth and infection by all pathogens, and the innate immune system withholds these metals from pathogens to limit their growth in a strategy termed “nutritional immunity.” While multimetal depletion by the host is appreciated, the majority of studies have focused on individual metals. Here, we use the innate immune protein calprotectin (CP), which complexes with several metals, including iron (Fe), zinc (Zn), and manganese (Mn), and the opportunistic pathogen Pseudomonas aeruginosa to investigate multimetal starvation. Using an unbiased label-free proteomics approach, we demonstrate that multimetal withholding by CP induces a regulatory response that is not merely additive of individual metal starvation responses, including the induction of lipid A modification proteins.

## INTRODUCTION

Transition metals are essential for all life, and invading microbial pathogens must acquire these nutrients to grow in the host and cause infection. The host innate immune system limits growth of microbial pathogens by withholding essential transition metals through a strategy termed “nutritional immunity” (1, 2). Metal-sequestering innate immune proteins are important components of this host response. Nutritional immunity originally focused on the competition for ferric iron [Fe(III)], wherein host proteins such as lactoferrin sequester Fe(III), and bacterial siderophores scavenge Fe(III) from these host proteins and deliver it to bacteria via siderophore receptors (1). Later, with the discoveries of additional metal-sequestering host proteins and microbial metal uptake systems, the model for nutritional immunity expanded to include other nutrient metals, such as manganese [Mn(II)] and zinc [Zn(II)] (2, 3). The S100 protein calprotectin (CP; S100A8/S100A9 oligomer, MRP8/MRP14 oligomer) plays a central role in nutritional immunity because it sequesters multiple divalent metal ions, including Mn(II), iron [Fe(II)], nickel [Ni(II)], and Zn(II) (reviewed in reference 4). CP exerts antimicrobial activity against a broad range of bacterial and fungal pathogens, and this activity is generally attributed to its metal-withholding ability (5–10).

Because the host deploys multiple metal-withholding proteins that coordinate various metal ions (e.g., CP, lactoferrin, siderocalin, and S100A12) at infection sites, bacterial pathogens must respond to the concerted limitation of multiple metal nutrients to cause infection (11). Moreover, bacterial pathogens exhibit various nutritional requirements for transition metals (e.g., some species have a high Fe requirement, whereas others require substantially more Mn [12, 13]) and are therefore likely to show species- and strain-specific responses to host multimetal withholding. Indeed, a recent study of Gram-negative and Gram-positive bacterial pathogens revealed that the effects of CP on the uptake of nutrient metals depend on the organism and the composition of the culture medium (10). Despite the current appreciation for multimetal sequestration by the host, the microbial response to this stress has received relatively less attention than single-metal limitation (11). Because CP sequesters multiple metals, it provides a physiologically important and useful tool for studying the response of microbial pathogens to multimetal withholding by the host.

Pseudomonas aeruginosa is a Gram-negative opportunistic pathogen that causes infections in vulnerable populations, including individuals with cystic fibrosis (CF). P. aeruginosa has a high Fe requirement and uses multiple Fe uptake systems to acquire Fe from the host environment, including siderophore-mediated Fe(III) uptake systems, an Fe(II) acquisition system, and heme uptake systems (reviewed in reference 14). Due to the propensity of excess Fe to cause oxidative damage, a complex and hierarchical Fe regulatory system regulates the uptake of Fe in response to cellular Fe concentrations (reviewed in reference 15). This function is mediated by the Fe-binding transcriptional regulator Fur, which in its holo form binds to the promoters of genes encoding Fe uptake systems, thereby blocking their transcription and Fe uptake (16). As Fe levels decrease, Fe dissociates from Fur, and apo-Fur loses affinity for these promoters, allowing transcription of Fur-repressed genes. In addition to Fe uptake genes, Fur also represses the PrrF small regulatory RNAs (sRNAs), which posttranscriptionally repress the expression of nonessential Fe-containing proteins and Fe storage proteins (17, 18). This so-called Fe-sparing response reduces the Fe requirement of the cell and is required for P. aeruginosa pathogenesis (19–21).

The mechanisms used by P. aeruginosa to maintain homeostasis of other transition metals are less well characterized, but several key aspects of select uptake and regulatory systems have been identified. The Zn(II) homeostasis system in P. aeruginosa includes Zur, a Fur homolog that represses the transcription of Zn(II) uptake systems (22). P. aeruginosa has several characterized Zn(II) uptake systems, including the Znu permease, the pseudopaline-metallophore-mediated Cnt (also called Zrm) system, and the HmtA P-type ATPase, as well as putative ABC permease gene clusters (PA2911-PA2914 and PA4063-PA4066) (23–26). Repression of Zn(II)-containing proteins has been shown under Zn(II)-limiting conditions, indicating a Zn(II)-sparing response (23, 26). When Zn(II) is in excess, transporters, such as the resistance-nodulation-division (RND) pump CzcCBA (27), the cation diffusion facilitator transporters CzcD and ZitB (28), and the P-type ATPase transporter ZntA (29), efflux Zn(II) to prevent toxicity. In terms of Mn homeostasis, P. aeruginosa encodes two predicted Mn(II) transporters (MntH1 and MntH2) (30) and requires Mn for several Mn-dependent proteins, including the ureohydrolases GbuA and GpuA (31) and the superoxide dismutase SodM (32). However, it remains unclear how this pathogen maintains Mn homeostasis. The copper (Cu) homeostasis system has also been characterized in P. aeruginosa and is composed of Cu importers, Cu chaperones, and Cu efflux pumps to maintain Cu homeostasis (33).

CP is found in high concentrations in the lungs of CF patients, which are commonly infected by P. aeruginosa (34–36). Moreover, CP has been shown to reduce the antimicrobial activity of P. aeruginosa toward another CF pathogen Staphylococcus aureus, which was attributed to its ability to inhibit the production of toxic secondary metabolites by P. aeruginosa, including phenazines and 2-alkyl-4(1*H*)-quinolones (AQs) (37). We previously reported, using targeted analyses of known Fe acquisition and regulatory systems, that CP withholds Fe from P. aeruginosa and thereby causes an Fe starvation response (10). Our study also demonstrated a change in virulence factor production in response to CP with the downregulation of phenazines, which we determined to be a consequence of Fe limitation (10). Moreover, a recent investigation demonstrated that CP withholds Zn(II) from P. aeruginosa and induces a Zn starvation response (38). This study also reported decreased Zn(II) protease activity caused by CP, indicating attenuated virulence potential. Because previous studies on the impact of CP on P. aeruginosa have focused on a single metal, we sought to determine the response of P. aeruginosa to multiple metal withholding by CP.

In this work, we investigated how CP-dependent metal depletion affects P. aeruginosa physiology and virulence capacity using quantitative, label-free proteomics. In agreement with prior studies (10, 38), our analyses show that CP causes both Fe and Zn starvation responses in P. aeruginosa. Moreover, we identified increases in the expression of Zn(II)-containing proteases and membrane-modifying enzymes in response to CP. To decipher whether the observed changes in protein expression resulted from single-metal or multimetal sequestration by CP, we used our proteomics workflow to evaluate the consequences of Fe, Mn, and Zn limitation on the P. aeruginosa proteome. This effort revealed that CP elicits an expected Zn starvation response and what appears to be an incomplete Fe starvation response. Moreover, CP treatment leads to the induction of membrane-modifying enzymes, likely as a consequence of multimetal sequestration. This response correlated with increased tolerance to polymyxin B, suggesting that host metal sequestration may induce P. aeruginosa tolerance to cationic antimicrobial peptides (CAMPs). Together, this work shows that the response of P. aeruginosa to multimetal withholding by CP is distinct from that of single-metal withholding and suggests a complex microbial response to this innate immune protein.

## RESULTS

### CP causes Fe and Zn starvation responses and alters virulence protein production in P. aeruginosa.

To determine the global response of P. aeruginosa to CP, we evaluated the impact of 10 μM CP treatment on the PA14 proteome. PA14 was grown under conditions that were previously used to study the consequences of CP treatment on P. aeruginosa and other pathogens (10, 39). Specifically, PA14 was cultured under aerobic conditions in a metal-replete chemically defined medium (CDM) containing 2 mM calcium (Ca), 5 μM Fe, 0.3 μM Mn, 6 μM Zn, 0.1 μM Ni, and 0.1 μM Cu in the presence or absence of 10 μM CP. These metal concentrations were selected to be within the reported range of metal levels in the sputa of CF patient samples (40–42), and the concentration of CP is within the range of CP concentrations in samples of sputa from CF patients (8 μM to 40 μM CP heterodimer) (43). We selected CDM for these studies because it allows for control over the metal concentrations and sources (e.g., nonheme versus heme Fe), and it is amino acid rich, similar to what is found in CF sputum (44). Prior whole-cell metal analyses in metal-replete CDM revealed that treatment of PA14 with CP results in a significant decrease in cell-associated Fe and negligible change in cell-associated Mn, Ni, Cu, and Zn (45). PA14 was grown for 8 h, which afforded growth to early stationary phase (10). Cells were harvested, and quantitative label-free proteomics was performed using nano ultraperformance liquid chromatography coupled to high-resolution tandem mass spectrometry as previously described (46–48). Protein levels that were significantly (*P* < 0.05, *n* = 5) changed at least 2-fold (equivalent to 1 log_2_ fold change [LFC]) were analyzed further.

**FIG 1:**
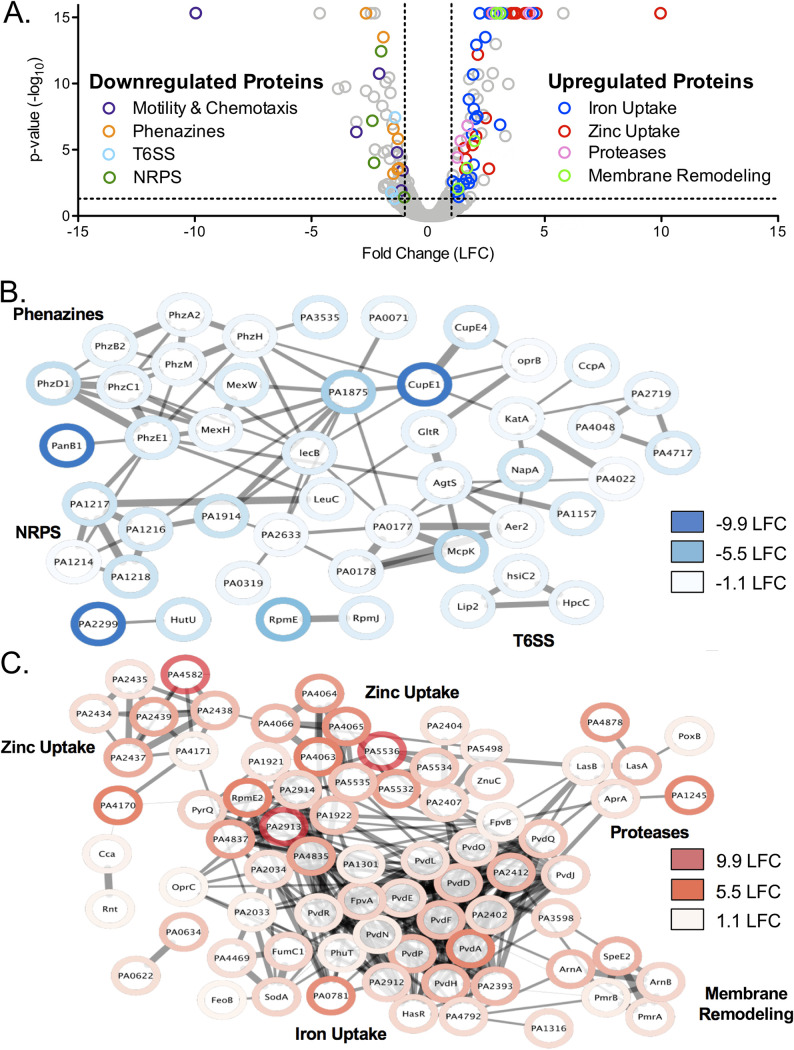
Proteomic analysis of P. aeruginosa response to CP. (A) Volcano plot of PA14 response to CP treatment. Every protein detected in the experiment is represented by an open, gray circle. Circles representing significantly regulated proteins in functional categories of interest are highlighted with the colors indicated in the legend. The thresholds for significance (*P* < 0.05) and expression (1 log_2_ fold change [LFC], equivalent to a 2-fold change) are marked by a black dashed line. Proteomics was performed with an *n* of 5, and significance was determined by an ANOVA test corrected for multiple testing by applying a Benjamini-Hochberg procedure (84). (B, C) Network analysis was performed using the STRING database (49) on the proteins that were significantly downregulated (B) and upregulated (C) in response to CP treatment. The thickness of lines between proteins indicates the strength of the data supporting the interaction, with more data correlating to a thicker line. The network was transferred into Cytoscape (79), and proteomics expression data were integrated using the Omics Visualizer app (80).

CP treatment caused a significant increase in the abundance of 93 proteins and a significant decrease in the abundance of 72 proteins (Fig. 1A; Table S1 in the supplemental material). To identify biological connections within the upregulated and downregulated proteins, we performed network analyses using the search tool for the retrieval of interacting genes/proteins (STRING) database (49), which generates a map to visualize the biological connections between proteins that were up- or downregulated. The proteins are represented by nodes and connected by edges, and the thickness of the edges represents the amount of associations based on known and predicted interactions, text mining, coexpression, and protein homology. The networks created for proteins that were downregulated (Fig. 1B; Table S1) or upregulated (Fig. 1C; Table S1) following CP treatment showed significantly more interactions than if they were a random collection of genes from the genome, indicating a biological relationship between the proteins in each analysis (Table S2). In agreement with recent studies showing that CP induces an Fe starvation response in P. aeruginosa (10, 45), proteins involved in pyoverdine-mediated Fe(III) uptake, in the Feo Fe(II)-import system, and in heme-dependent Fe acquisition were upregulated in response to CP, whereas proteins involved in phenazine biosynthesis (PhzE, PhzD, PhzC, PhzM, and PhzH) and secretion (MexH and MexI) were downregulated (Fig. S1). CP treatment also resulted in an apparent Fe-sparing response, indicated by the downregulation of nonessential Fe-containing proteins. For example, nonessential Fe- and heme-containing proteins, such as LeuC and KatA, were downregulated upon CP treatment, whereas the Fe-independent paralog of fumarase (FumC1) and the Mn-dependent superoxide dismutase (SodM) were upregulated in response to CP (Fig. S1). However, CP treatment did not reduce levels of several Fe-containing tricarboxylic acid (TCA) cycle proteins, which were previously shown to be repressed by the PrrF sRNAs as a part of the P. aeruginosa Fe-sparing response (17, 18, 50, 51).

Consistent with a recent study (38), CP-treated cells showed a robust Zn starvation response. This response included increased levels of proteins for Zn(II) uptake systems, including ZnuACD and proteins for the synthesis and uptake of pseudopaline (CntM and CntO, respectively) (22, 25, 26). CP treatment also led to an increase in several predicted Zn(II) uptake proteins (PA4063-PA4066, PA2912-PA2914, PA1921, and PA1922) that were shown in a previous transcriptomic study to be induced by Zn starvation (25, 26). Also consistent with the previously described Zn starvation response (23), Zn(II)-containing proteins RpmE and RpmJ were downregulated, and Zn(II)-independent proteins RpmE2, DksA2, and PyrC2 were upregulated following CP treatment (Fig. S3). Despite the robust Zn starvation response to CP observed here and in previous work (38), only negligible changes in cell-associated Zn levels were observed for PAO1 and PA14 grown in metal-replete CDM supplemented with 10 μM CP (10, 45).

Although CP has the ability to withhold Mn, levels of known Mn cofactored proteins (HutG, GpmI, and UbiD) and predicted Mn uptake proteins (MntH1 and MntH2) in PA14 were not significantly affected by CP treatment. Prior work revealed that the ability of CP to reduce cell-associated Mn in P. aeruginosa is medium dependent (10, 45). Whereas CP treatment reduced cell-associated Mn levels when PA14 and PAO1 were grown in a mixture of Tris buffer and tryptic soy both (Tris:TSB) (10), a negligible change in cell-associated Mn was observed when these strains were treated with CP in metal-replete CDM (45). This medium effect may explain the lack of a putative Mn starvation response in this proteomics analysis. As noted above, the Mn-dependent superoxide dismutase SodM was upregulated in response to CP, possibly resulting from an Fe starvation response.

In addition to known Fe- and Zn-responsive proteins, proteins for the type VI secretion system (T6SS) (Lip2.1, HsiC2, and HcpC), motility and chemotaxis (Aer, Chew2, CheA2, FlgA, CupE2, and CupE4), and a nonribosomal peptide synthetase (NRPS) of unknown function (PA1214, PA1216, PA1217, and PA1218) were downregulated following CP treatment (Fig. 1; Table S1). CP treatment also led to the upregulation of proteins involved in membrane remodeling and tolerance to CAMPs (PmrA, PmrB, ArnA, ArnB, and SpeE2). Curiously, CP treatment caused an increase in several Zn(II)-dependent metalloproteases (ImpA, AprA, LasA, and LasB), contrasting with the Zn-sparing response described above. Altogether, this proteomics analysis indicates that CP exposure induces both Fe and Zn starvation responses by P. aeruginosa and alters the expression of proteins involved in several virulence processes.

### CP elicits Fe and Zn starvation responses that are distinct from single-metal limitation.

We next evaluated whether the changes in protein expression described above for CP-treated PA14 resulted from sequestration of an individual metal, multimetal sequestration, or a metal-independent response to CP using the same quantitative proteomics workflow. We focused these analyses on the limitation of Mn, Fe, and Zn since the ability of CP to sequester Mn(II), Fe(II), and Zn(II) and induce microbial responses to the limitation of these metal nutrients is established (7, 10, 52). For these experiments, CDM was prepared in the absence of either Fe, Mn, or Zn or was lacking all three metals to afford “Fe-depleted,” “Zn-depleted,” “Mn-depleted”, and “metal-depleted” CDM, respectively, and protein abundance was compared to that of PA14 grown in metal-replete CDM. A network analysis was used as described above to determine biological connections within the upregulated and downregulated proteomes in response to Fe, Zn, and Mn limitation (Table S1).

The Fe starvation response observed following growth in Fe-depleted CDM was consistent with the well-known regulatory response of P. aeruginosa to Fe limitation (17, 50, 51) (Fig. S1), despite the use of a lower Fe concentration in the metal-replete condition (5 μM instead of 100 μM), a chemically defined medium (CDM instead of Chelex-treated and dialyzed TSB [DTSB]), a different time point (early instead of late stationary phase), and a different strain of P. aeruginosa (PA14 versus PAO1). Specifically, Fe limitation resulted in the downregulation of Fe-containing TCA cycle enzymes (SdhBAC, AcnA, PA0794, and PA4330), a putative bacterioferritin (PA4880), and Fe-containing oxidative stress response proteins (SodB and KatA), the upregulation of Fe-independent homologs of TCA cycle enzymes (MqoA and FumC1) and a superoxide dismutase (SodM), and the upregulation of pyoverdine- and heme-dependent Fe uptake systems. When PA14 was grown in Zn-depleted CDM, a Zn starvation response was observed that was consistent with the transcriptomic response to Zn limitation and investigation of Zn(II) uptake mutants described in previous investigations (Fig. S2) (25, 26). Specifically, we observed the downregulation of Zn(II)-containing proteins (RpmE and RpmJ) and the upregulation of Zn(II)-independent paralogs of Zn(II)-containing proteins (RpmE2, DksA2, PyrC2, CynT2, and FolE2). CP treatment also led to upregulation of proteins for the biosynthesis and transport of pseudopaline (CmtM, CmtL, and CmtO), ZnuABC, the Zn(II)-responsive HmtA heavy metal transport system, and several predicted Zn(II) uptake systems (PA4063-PA4066, PA2912-2914, and PA1921). These results demonstrate that the growth conditions used here and in previous studies to investigate CP (10) allow for characteristic P. aeruginosa Fe and Zn starvation responses.

When PA14 was grown in Mn-depleted CDM, we found no evidence for a putative Mn starvation response. The predicted Mn(II) transporters MntH1 and MntH2 were not significantly upregulated in Mn-depleted CDM, and neither known nor predicted Mn-containing proteins were downregulated beyond the established LFC threshold (30). We noted a significant downregulation of SodM, although the fold change in this protein was below our threshold of 1 LFC (0.8 LFC). Additionally, there was no significant biological connection between the upregulated or downregulated proteins identified by network analysis (Table S2). Because no robust Mn starvation response was observed following either Mn limitation or CP treatment, we focused the remainder of our metal depletion studies on Fe and Zn.

**FIG 2:**
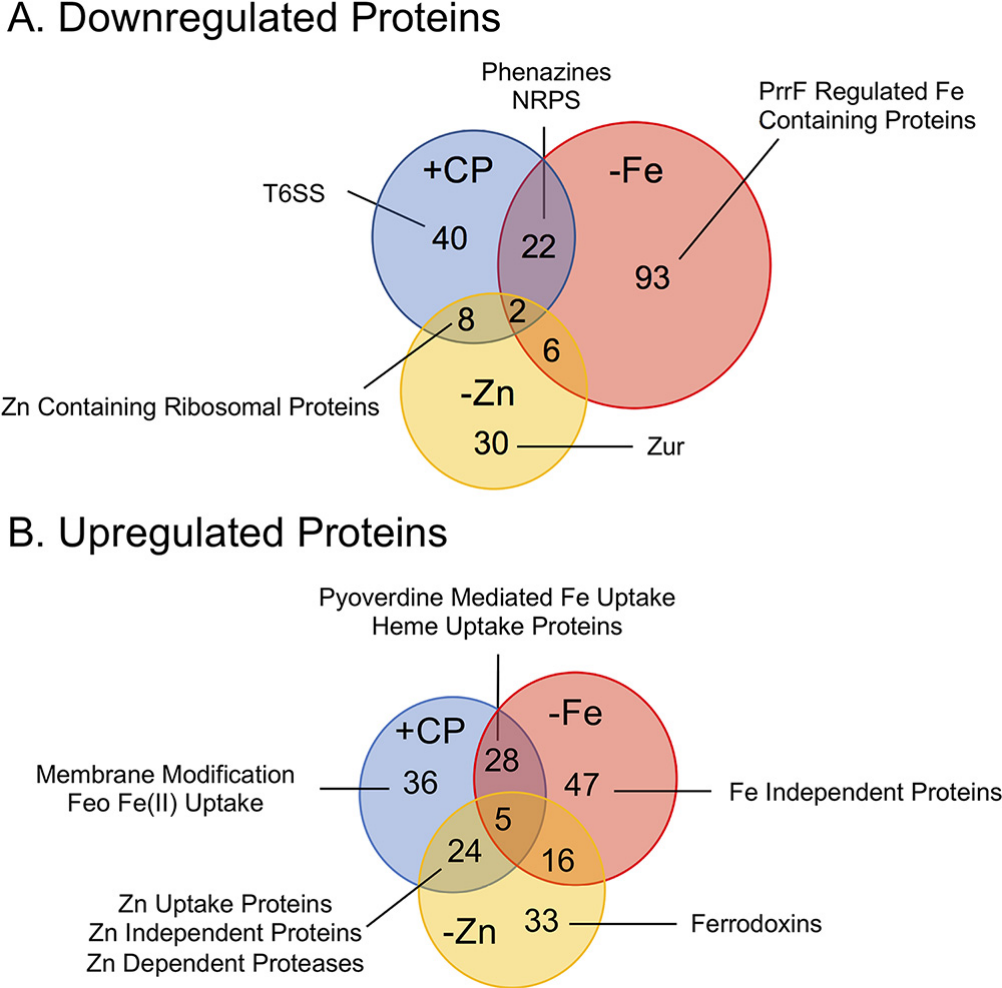
Comparison of the proteome in response to Fe and Zn limitation and CP treatment. Venn diagrams of significantly (*P* < 0.05) downregulated (>1 LFC) (A) and upregulated (>1 LFC) (B) proteins in response to CP treatment (+CP), Fe limitation (−Fe), and Zn limitation (−Zn). Proteomics was performed with an *n* of 5; LFC, log_2_ fold change.

We next identified overlaps in the proteomic responses to CP treatment, Fe limitation, and Zn limitation. We observed an ∼35% overlap between proteins upregulated by CP treatment and Fe limitation, including proteins involved in the uptake of Fe(III)-pyoverdine and heme (Fig. 2B; Table S3). Proteins that were observed to be upregulated by Fe limitation but not CP treatment included several Fe(III) uptake proteins that were either not detected in the CP treatment experiment (8 proteins) or not significantly upregulated beyond the 1 LFC threshold (PhuR). Approximately 20% of the proteins that were downregulated in response to Fe limitation were also downregulated in response to CP (Fig. 2; Table S3). These proteins included phenazine biosynthesis and secretion proteins and the NRPS operon noted above. Further analysis of the 99 downregulated proteins that appeared specific to Fe limitation revealed that 9 of these proteins were significantly (*P* < 0.05) downregulated following CP treatment, but the change did not meet the 1 LFC threshold, possibly indicating a weaker Fe starvation response to CP than induced by Fe-depleted CDM (Fig. S1). Notably, several PrrF regulon proteins, including TCA cycle enzymes (SdhABCD and AcnB), NADH dehydrogenase (NuoABCEFGHI), and the putative bacterioferritin (PA4880), were not significantly changed in the presence of CP, indicating that CP treatment elicits an incomplete Fe-sparing response. Previous studies using an AntR transcriptional and translational reporter strain showed that AntR, which is downregulated by PrrF during growth in DTSB (53), is also downregulated in response to CP treatment during growth in Tris:TSB (10). However, AntR and its regulatory targets AntABC were not detected in this proteomics experiment. To determine whether the Fe starvation response to CP during growth in metal-replete CDM included a decrease in AntR, we used the same reporter strain and found that *antR* expression is similarly repressed by CP treatment during growth in metal-replete CDM (Fig. S2), indicating that PrrF functions under these conditions. Combined, these data indicate that that CP elicits only a portion of the Fe starvation response that is observed following Fe limitation in Fe-depleted CDM.

The shared response to both Zn limitation and CP treatment was consistent with the known Zn starvation response and included the downregulation of Zn(II)-containing ribosomal proteins (RpmJ and RpmE) (Fig. 2A; Fig. S3) and the upregulation of Znu transport proteins (ZnuA and ZnuC), pseudopaline biosynthesis and transport (CntM and CntO) proteins, the Zn(II)-independent ribosomal protein RpmE2, and the Zn(II)-independent paralog to DksA, DksA2 (Fig. 2B; Fig. S3). Notably, several Zn(II)-dependent proteases (LasA, LasB, ImpA, and AprA) were upregulated by both CP and Zn(II) limitation, seemingly contrary to the observed Zn starvation response and a recent report on decreased protease activity in response to CP (38) (Fig. 2B). The upregulated proteins that were specific to the Zn limitation proteome included Zn(II)-independent proteins (CynT2 and FolE2) that were not significantly changed in response to CP. Moreover, two ferredoxins (Fdx2 and FdxA) were upregulated by Zn limitation and were not significantly affected by CP treatment (Table S3). Notably, the Zn(II)-responsive transcriptional regulator Zur was downregulated only in response to Zn limitation. Previous work demonstrated that *zur* is cotranscribed with *znuC* and *znuB*, and expression of all three genes is induced by Zn limitation (Fig. S4A) (22). However, in the current work, Zur was downregulated by Zn limitation and unchanged in response to CP, whereas ZnuC and ZnuB were upregulated in both the Zn-depleted and CP treatment conditions (Fig. S4B). We performed real-time PCR (RT-PCR) analysis to investigate this finding further and observed that expression of both the *znuA* and *zur* mRNAs were induced by CP treatment and by Zn limitation (Fig. S4C and D), suggesting that Zur is posttranscriptionally downregulated following Zn limitation and CP treatment. Together, these observations suggest that the response of P. aeruginosa to Zn starvation is more complex than currently appreciated; this notion warrants further investigation.

**FIG 3:**
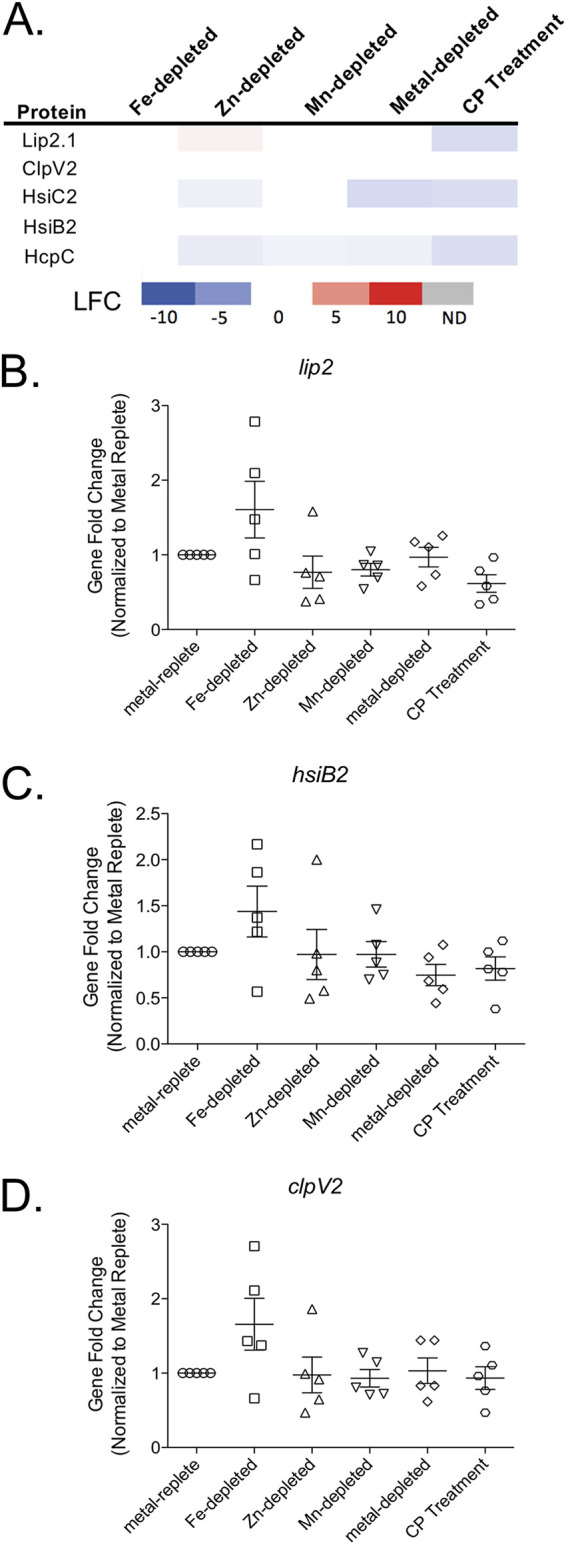
T6SS proteins are downregulated in response to CP. (A) Heat map of protein expression of P. aeruginosa PA14 grown in Fe-depleted, Zn-depleted, Mn-depleted, metal-depleted CDM, and metal-replete CDM in the presence of CP compared to expression in metal-replete CDM. The log_2_ fold change (LFC) is shown for all significantly (*P* < 0.05) changed proteins. Gene expression under the same conditions was measured for *lip2* (B), *hsiB2* (C), and *clpV2* (D) using RT-PCR. No significance was detected in any of the comparisons. Significance was determined by one-way ANOVA with Dunnett’s multiple-comparison test (*n* = 5).

The comparisons between the responses of PA14 to CP, Fe limitation, and Zn limitation also identified responses that were unique to CP treatment. Several T6SS proteins encoded by the HSI-II T6SS locus (Fig. 2A and 3A) were downregulated only in response to CP. This result was surprising given that our recent proteomic studies showed that Fe starvation upregulates the expression of the same T6SS proteins (46). However, the mRNAs encoding three of the HSI-II T6SS proteins (*lip2*, *clipV2*, and *hsiB2*) were not significantly changed in a subsequent RT-PCR experiment (Fig. 3B to D), contrasting with a recent Fe regulation study (46) and the current proteomics results (Fig. 3A). The difference in gene expression between this work and the previous study may result from differences in experimental conditions, and posttranscriptional effects may be responsible for the distinct effects of CP on protein expression. Also notable was the Feo Fe(II) import system, which was upregulated only in response to CP but not in response to Fe limitation (Fig. 2A; Fig. S1). Additionally, proteins involved in membrane modification and CAMP tolerance (PmrA, PmrB, ArnA, ArnB, and SpeE2) were upregulated only in response to CP (Fig. 2B; Table S3). The Feo Fe(II) transport system has been shown to be regulated by PmrAB (54), possibly explaining the increase in its expression in response to CP. Together, these data indicate that CP treatment elicits a physiological response that overlaps with, but is distinct from, the P. aeruginosa Fe and Zn starvation responses.

### CP treatment and Zn limitation reduces LasB activity despite increases in protease levels.

**FIG 4:**
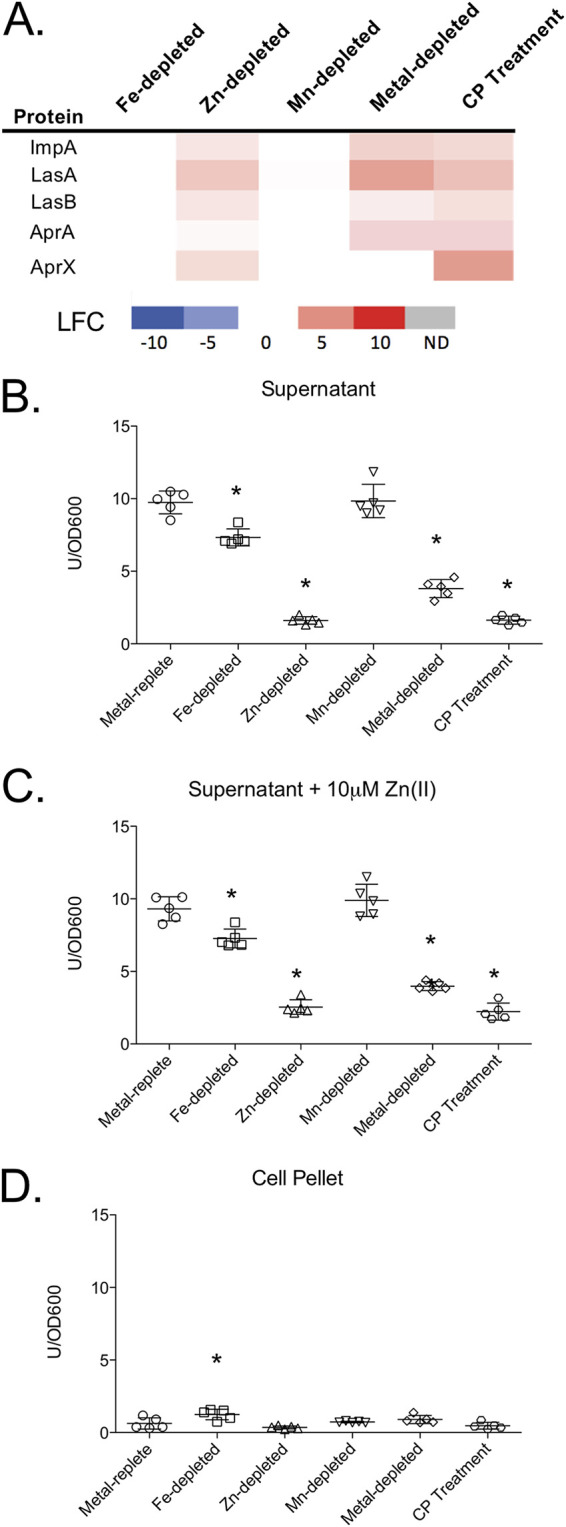
LasB activity decreased in response to Zn limitation despite an increase in cell-associated protein abundance. Heat map (A) of secreted proteases shown for all experimental conditions compared to the metal replete control. The log_2_ fold change (LFC) of significantly (*P* < 0.05) changed proteins is shown for selected proteins. LasB activity was measured in the supernatant in the absence (B) and presence (C) of 10 μM Zn(II) in the assay buffer to determine if activity could be restored. LasB activity of the cell lysate (D) was also determined. Activity was determined by azocasein assay and normalized to OD_600_ after 8 h of growth. Significance was determined by one-way ANOVA with Dunnett’s multiple-comparison test; *n* = 5; *, *P* < 0.05.

The observed increase in the levels of secreted proteases (LasB, LasA, AprA, and ImpA) in response to Zn limitation and CP treatment (Fig. 4A) was surprising for several reasons. First, these proteases are secreted proteins, and our workflow was designed to analyze the cell-associated proteomes of P. aeruginosa. Second, a previous study demonstrated decreased LasB activity under Zn-limiting conditions (26), and more recent work showed a similar decrease in protease activity upon chelation of Zn(II) by CP (38). To investigate this finding further, we quantified LasB activity against azocasein of PA14 grown in metal-replete, Fe-depleted, Zn-depleted, Mn-depleted, and metal-depleted CDM or in the presence of CP. In contrast to what was observed with cell-associated protease levels and in agreement with previous studies (26, 38), LasB activity in the culture supernatants decreased following both Zn limitation and CP treatment (Fig. 4B). A previous study with PAO1 reported the restoration of LasB activity in Δ*znuA* and Δ*cntO* Zn(II) uptake mutants with the addition of Zn(II) to the assay buffer but not in Δ*znuA*Δ*cntO* mutants (26). Similar to the Δ*znuA*Δ*cntO* mutant, we were unable to recover LasB activity to Zn-depleted or CP-treated culture supernatants by supplementing the assay buffer with Zn(II) (Fig. 4C). The Δ*znuA*Δ*cntO* double mutant had a marked growth defect during growth in minimal medium and significantly lower cell-associated Zn than wild-type cells and single mutants, indicating that it was more Zn starved than the single mutants (26). One possible explanation is that the Zn-depleted and CP-treated cultures were similarly too Zn starved to recover protease activity with the addition of Zn(II) to the assay buffer. An alternative explanation is that, under our culture conditions, the proteases were not secreted, which is supported by the increase in these proteins in the cell-associated proteome.

To determine if the decrease in secreted protease activity following Zn limitation or CP treatment was due to decreased secretion of the proteases, we determined whether a corresponding increase in LasB activity was observed in these cell lysates. We noted a small but statistically significant increase in protease activity in cell lysates from the Fe-depleted condition compared to lysates from the metal-replete condition (Fig. 4D). However, no change was noted in lysates from the Zn limitation or CP treatment conditions. There is currently some debate in the literature on whether LasB is secreted in its inactive proform or activated in the periplasm, but a recent study indicates that LasB is activated after secretion (55). Thus, these data do not necessarily refute the hypothesis that decreased protease activity is due to decreased secretion.

To further investigate the hypothesis that secretion of proteases is decreased in response to Zn limitation, we considered that the expression of secretory systems responsible for export of each of these proteases may be downregulated under these conditions. LasA, LasB, and ImpA are secreted by the Xcp and Hxc type II secretion systems (T2SS) (56, 57), whereas the alkaline protease AprA is secreted by a type I secretion system (T1SS) encoded by the *aprDEF* operon upstream of the *aprA* gene (58). Among the proteins for each of these systems, our proteomics data showed that only two proteins (XcpX and AprF) were downregulated following CP treatment. Upon Zn limitation, XcpX was not significantly changed, and AprF was downregulated below our threshold of 1 LFC (Fig. S5A). In contrast, HxcV was strongly repressed when PA14 was cultured in Zn-depleted medium. This protein was not detected in the CP-treated samples; thus, we cannot exclude the possibility that CP similarly repressed its expression. Further gene expression analysis showed that expression of *xcpT* did not change following CP treatment or Zn limitation, whereas *xcpP* expression increased a small but statistically significant amount in response to both CP treatment and Fe limitation (Fig. S5B and C). Similarly, expression of genes within the *xcp* operons was not significantly changed under any condition tested (Fig. S5D to G). Overall, these data suggest that decreased expression of proteins that secrete Zn(II)-dependent proteases is unlikely to contribute to the decreased LasB activity in culture supernatants despite increased levels of these proteins within cells.

### CP treatment increases levels of membrane-remodeling proteins.

**FIG 5:**
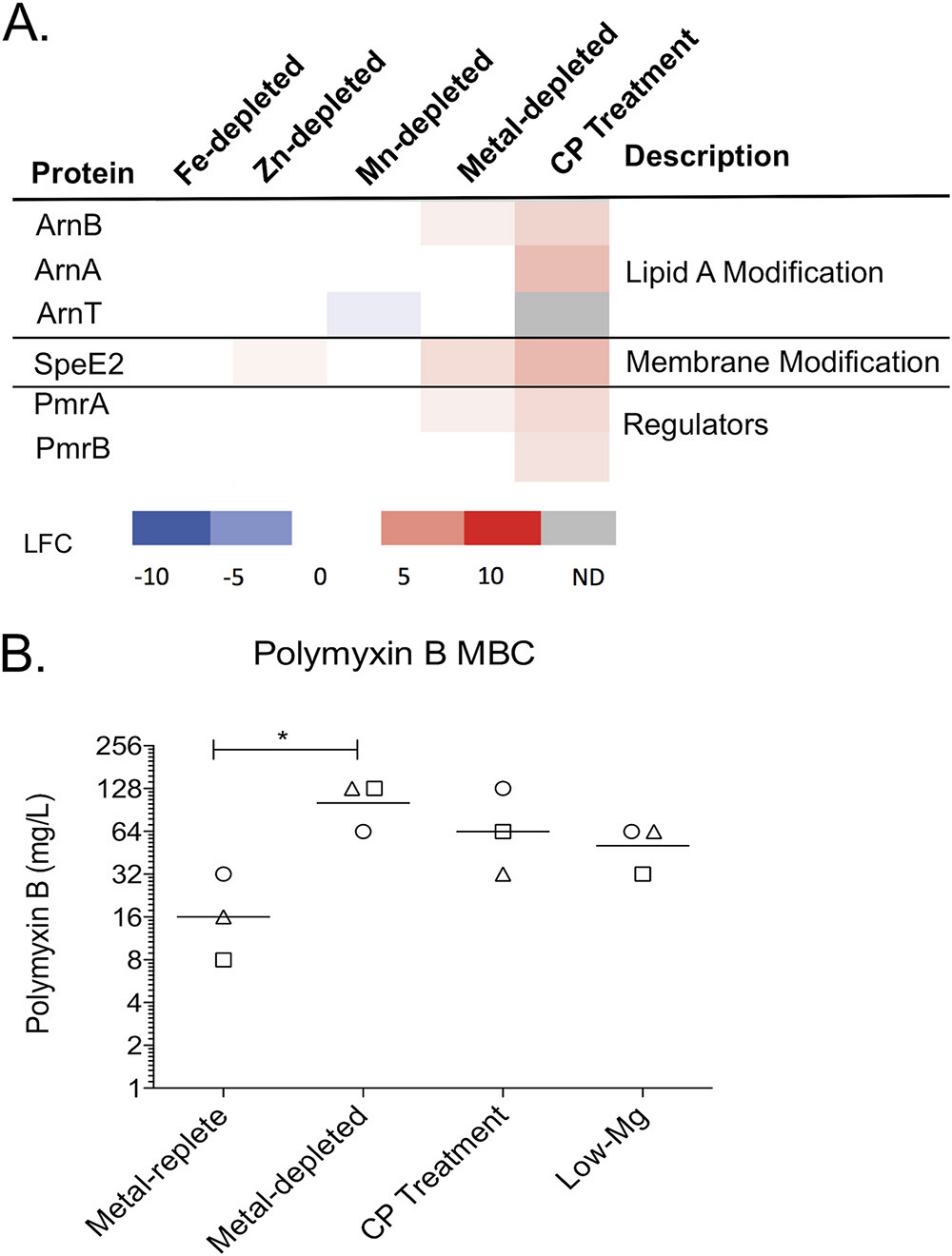
Polymyxin B tolerance increases after exposure to CP. (A) Heat map of membrane-remodeling proteins under all metal depletion conditions. Protein expression is compared to the metal-replete condition. The log_2_ fold change (LFC) of each significantly (*P* < 0.05) changed protein is shown. (B) The minimal bactericidal concentration (MBC) of polymyxin B was determined for PA14 after preculture in metal-replete, metal-depleted, and low-Mg(II) CDM and after CP treatment. The geometric mean of the three biological replicates for each condition is denoted with a line. The biological replicates are represented with circles (replicate 1), squares (replicate 2), and triangles (replicate 3). Significance was determined by one-way ANOVA with Dunnett’s posttest; *, *P* < 0.05.

As mentioned above, CP treatment resulted in increased expression of the two-component regulator PmrAB and its regulatory targets ArnA and ArnB, which synthesize aminoarabinose and the spermidine synthetase SpeE2 (Fig. 5A) (59, 60). The addition of spermidine to lipopolysaccharide (LPS) and aminoarabinose to lipid A promotes tolerance to CAMPs, including colistin (polymyxin E) and polymyxin B (59–62). The literature refers to an increase in resistance to CAMPs; however, the change in susceptibility is transient and in response to the environment, hence, we have used tolerance (63). Initially, it appeared that the induction of membrane-remodeling machinery did not overlap with the Fe or Zn starvation responses and therefore may be part of a metal-independent response. Further analysis, however, showed that Zn limitation caused a small but significant increase in SpeE2 that fell below our initial LFC threshold of 1 (0.6 LFC). Moreover, SpeE2, ArnB, and PmrA were significantly upregulated in metal-depleted CDM, suggesting that this CP-induced response resulted from multimetal sequestration. To investigate whether CP treatment afforded CAMP tolerance in P. aeruginosa, we evaluated how CP pretreatment of PA14 affected the minimal bactericidal concentration (MBC) of polymyxin B. Because previous studies demonstrated increased polymyxin B tolerance after growth under low-Mg(II) conditions that results from aminoarabinose modification of lipid A (54), PA14 was also precultured in metal-replete CDM supplemented with 20 μM Mg(II) (low-Mg CDM), instead of the standard 2 mM Mg(II), as a positive control. Although we noted some variation in MBCs between three biological replicates, bacteria precultured in metal-replete CDM were consistently less tolerant to polymyxin B than bacteria precultured in metal-replete, low-Mg CDM (Fig. 5B; Table S4). PA14 precultured in either metal-depleted CDM or pretreated with CP exhibited MBC ranges (64 to 128 mg/liter and 32 to 128 mg/liter, respectively) that overlapped with that of the cultures grown in low-Mg CDM (32 to 64 mg/liter). Statistical significance (*P* < 0.05) was identified between the metal-depleted and metal-replete samples by one-way analysis of variance (ANOVA) with a Dunnett’s posttest. More in-depth investigations of this finding are under way. Together, these data suggest that metal withholding and CP increases the tolerance of PA14 to polymyxin B.

## DISCUSSION

In the current work, we systematically evaluated the contributions of Fe, Zn, and Mn limitation to the proteomic response of P. aeruginosa to CP in a chemically defined medium. We previously demonstrated an Fe starvation response to CP (10) but reasoned that a broader physiological response was necessary to maintain metal homeostasis in response to CP multimetal withholding. Our results demonstrate that the response of P. aeruginosa to CP is not simply an additive response to Fe and Zn withholding. Moreover, we identified CP-dependent induction of membrane modification proteins that appear to afford polymyxin B tolerance. Because this response was not observed following depletion of any single metal, we hypothesize that it is dependent on a general depletion of transition metals by CP. Consequently, this work demonstrates that multimetal sequestration elicits distinct effects compared to individual metal depletion on the physiology and virulence capacity of a human pathogen.

A goal of our study was to determine how sequestration of Fe, Zn, and Mn contributes to the response of P. aeruginosa to CP. Thus, in our analyses, specific responses to single-metal limitation were compared to the response to CP multimetal withholding. This analysis revealed that the CP-induced Fe starvation response was markedly incomplete compared to the Fe starvation response observed in Fe-depleted medium. For example, CP induced upregulation of Fe acquisition proteins; but, many Fe-containing metabolic proteins, which are normally downregulated by the PrrF sRNAs upon Fe limitation (51), were not significantly affected by CP treatment. Consistent with our findings, a previous transcriptomic study of the response of P. aeruginosa to CP showed decreased expression of phenazine biosynthesis genes and increased expression of pyoverdine biosynthesis genes but no change in the expression of PrrF-responsive TCA cycle genes (37). This earlier study attributed many of these changes to Zn limitation because the Fe(II)-withholding ability of CP was not yet accepted, leading to the proposal that a subset of Fe-regulated genes could also be responsive to other metals (37). Nevertheless, our previous proteomics study of Fe-limited PAO1 demonstrated that decreased expression of phenazine biosynthesis proteins and increased expression of pyoverdine biosynthesis proteins occurred in a strictly Fe-dependent manner, suggesting that CP induces some, but not all, of the prototypical Fe starvation responses in P. aeruginosa (10). The current study further supports that decreased expression of phenazine biosynthesis proteins and increased expression of pyoverdine biosynthesis proteins result from Fe limitation. The origin or origins of the incomplete Fe starvation response to CP are unclear. We currently speculate that it results from a tiered response to different levels of Fe limitation similar to what was found for the Bacillus subtilis Zn starvation response (64). For example, the Zn starvation response that is elicited by CP treatment may mask the activation of specific Fe starvation pathways, resulting in a distinct Fe starvation response following CP treatment compared to Fe limitation. More work is needed to understand the observed differences in Fe starvation responses between these two conditions.

We also observed a robust Zn starvation response to CP, evidenced by the upregulation of multiple Zn(II) uptake systems by PA14. In contrast to the incomplete CP-induced Fe starvation response described above, the Zn starvation response elicited by CP was comparable to that caused by Zn limitation, suggesting a robust response to Zn(II) withholding by CP. Notably, the Zn starvation response is induced in the absence of a decrease in cell-associated Zn during CP treatment (10, 45). This observation is consistent with previous reports that CP treatment results in metal starvation responses without changes to the corresponding cell-associated metal concentrations (65, 66). For instance, CP was found to exert antimicrobial activity and induce a Zn starvation response in Candida albicans without decreasing cell-associated Zn (65). In Acinetobacter baumannii, the Cu uptake protein OprC was downregulated, and a Cu storage protein was upregulated, indicating a Cu starvation response to CP without a change in cell-associated Cu (66). One reason for these differences is that the measurements provide the total cellular metal content, which includes both labile metal pools that can be sensed by the cell as well as kinetically inaccessible (e.g., stored) metal pools. Together, these data indicate that a decrease in cell-associated metal content is not always a reliable indicator of how pathogens respond to multimetal withholding.

In addition to the Zn starvation response, CP treatment and Zn limitation both resulted in an unexpected increase in Zn(II)-dependent proteases. Further analyses showed that CP treatment and Zn limitation decreased LasB activity in culture supernatants, aligning with previous studies (24, 26, 38, 67). Because of the intracellular increase in abundance of the secreted proteins, we initially hypothesized that a decrease in protease secretion may contribute to the observed decrease in LasB activity. However, we observed no increase in LasB activity in the cell lysate and little change in protein abundance or in transcript levels for the Xcp and Hxc T2SS systems that export these proteases, although it is possible that protease secretion is reduced by an unknown mechanism. A previous study demonstrated that CP chelated Zn(II) from proteases when the supernatant was treated with CP (38). Further work will be needed to determine the mechanism of decreased protease during Zn limitation and to determine the mechanism of decreased protease activity during CP treatment of cultures.

Recently, CP was shown to induce a multimetal starvation response in A. baumannii (66). In addition to the induction of siderophores and other Fe uptake systems, the authors proposed a dual regulatory system that responds to Zn withholding and flavin mononucleotide (FMN) concentration to induce a Zn(II)-independent enzyme that maintains flavin biosynthesis during Zn limitation. Flavin biosynthesis is necessary for the production of flavodoxins and FMNs, which can act as Fe-independent substitutes for ferredoxins and cofactor Fe-independent homologs, respectively. Our recent work identified potential crosstalk between the Fe and Zn homeostasis systems of P. aeruginosa (51), but this crosstalk was not evident in the current study, possibly due to the 20-fold lower Fe concentration used for the proteomics experiments. Thus, it remains unclear how much the interactions of distinct metal regulatory systems contribute to the response of P. aeruginosa to CP.

We also identified what appears to be a multimetal limitation response following CP treatment with the induction of the PmrAB two-component regulatory system and its targets ArnA, ArnB, and SpeE2 (Fig. 2 and 5). PmrAB activation of the *arn* operon results in the addition of aminoarabinose modifications to lipid A, resulting in increased tolerance to CAMPs, such as colistin and polymyxin B. The PmrAB regulon is known to be induced by Mg(II) and Ca(II) limitation (59, 68). Here, we provide evidence that this operon is more generally responsive to low concentrations of transition metal limitation caused by CP. A previous study demonstrated that extracellular DNA binds cations and induces resistance to CAMPs through the activity of PmrAB-regulated proteins (69), further supporting the conclusion that a more generalized low-cation environment can initiate the PmrAB pathway. We provide evidence that both CP treatment and transition metal depletion results in increased tolerance to polymyxin B. While we did not address the mechanism in this study, the observed upregulation of ArnA and ArnB suggests that increased tolerance occurs through the addition of aminoarabinose to lipid A (59, 61). This modification is highly relevant in the clinical setting as aminoarabinose modification of lipid A is commonly observed in polymyxin-tolerant P. aeruginosa isolates (70, 71). Nebulized colistin is commonly used to reduce P. aeruginosa burdens in the lungs of CF patients (72–74). Our results suggest that CP released during the inflammatory response in the CF lung may promote P. aeruginosa to tolerate this treatment. CP also decreased activity of lipid A biosynthesis proteins in Helicobacter pylori, resulting in altered lipid A structure, which led to increased biofilm production and reduced growth inhibitory activity of CP (75). It is possible that changes to lipid A are a widespread response of Gram-negative bacteria to CP.

In closing, the results from this proteomics study provide new insights into host-pathogen interactions by systematically evaluating how multimetal withholding by CP impacts P. aeruginosa physiology. This work demonstrates that the response of P. aeruginosa to metal withholding by CP is not the additive response of individual metal starvation responses. Moreover, metal starvation responses are linked to virulence regulatory networks in many pathogens, and the current work highlights that response of P. aeruginosa to multimetal limitation elicits several changes in virulence-related processes. Thus, this investigation broadens our understanding of how nutritional immunity impacts the pathogenic potential of an important opportunistic bacterial pathogen.

## MATERIALS AND METHODS

### Growth media and conditions.

P. aeruginosa strain PA14 was used for all experiments. Throughout the paper, we have used the locus tags for PAO1 instead of PA14 when gene names are not available for ease of reading and comparison to the literature. PA14 was grown as previously described in chemically defined medium (CDM). CDM was made as previously described (10, 39) with some modifications (Table S4 in the supplemental material). Briefly, the medium was made without added Mg(II) and was then aliquoted and stored at –80°C. Before an experiment, the medium was thawed and supplemented with 0.1 μM NiCl_2_, 0.1 μM CuCl_2_, 5 μM FeSO_4_, 6 μM ZnCl_2_, 0.3 μM MnCl_2_, 2 mM MgSO_4_, and 2 mM CaCl_2_ to afford metal-replete CDM. Calprotectin (CP; 10 μM final concentration) was added to CDM where indicated. Metal-depleted CDM was made by supplementing with only NiCl_2_, CuCl_2_, MgSO_4_, and CaCl_2_. Fe-depleted, Zn-depleted, and Mn-depleted CDM were made by not adding Fe, Zn, or Mn, respectively, to the CDM. Low-Mg CDM was made by adding 20 μM MgSO_4_ instead of 2 mM MgSO_4_ to metal-replete CDM. Cultures for all experiments were inoculated to an optical density at 600 nm (OD_600_) of 0.05 and grown for 8 h with shaking at 250 rpm at 37°C. Except for the proteomics experiment, all experiments were repeated at least three times to ensure reproducibility.

### Calprotectin purification.

CP was purified as previously described (76). Protein aliquots were stored in 20 mM HEPES, 100 mM NaCl, and 5 mM dithiothreitol (DTT) (pH 8.0) at –80°C. Aliquots were thawed only once before use and buffer exchanged three times into 20 mM Tris and 100 mM NaCl (pH 7.5) using presterilized 10 kDa molecular weight cutoff (MWCO) spin concentrators (Amicon). Protein concentrations were determined by *A*_280_ using the calculated extinction coefficient of the CP heterodimer (*ε*_280_ = 18,540 M^−1^cm^−1^) obtained from the online ExPASy ProtParam tool.

### Quantitative label-free proteomics.

Two independent proteomics experiments were performed each with five biological replicates. The medium for both experiments was prepared as described above as a single batch and inoculated with the same five overnight cultures, and samples were collected for both experiments at the same time to limit variability. For the first experiment, PA14 was grown in metal-replete CDM with and without 10 μM CP. For the second experiment, PA14 was grown in metal-replete, Fe-depleted, Mn-depleted, Zn-depleted, and metal-depleted CDM. Quantitative label-free proteomics was performed using a Waters nanoACQUITY ultraperformance liquid chromatography (UPLC) system coupled to a Thermo Orbitrap Fusion Lumos Tribrid mass spectrometer similar to as previously described with modifications (46–48). A full description of the mass spectrometry-based experimental methodology is provided in the supplemental materials. Gene function and pathway analysis was conducted using information from the Pseudomonas genome database (77), the Pseudomonas metabolome database (78), and the STRING database (49).

### Network analysis.

Network analysis was performed using the STRING database, version 10.5 (49). Corresponding PAO1 accession numbers were used as the database is limited to the PAO1 strain. The network was downloaded from STRING and further analyzed using Cytoscape (79). The Omics Viewer app was used within Cytoscape to incorporate proteomics expression data into networks (80).

### AntR reporter assay.

PA14/P*antR*-′*lacZ^−^*^SD^ was generated previously (10). Cultures were grown in metal-replete CDM with or without 10 μM CP as described above. β-Galactosidase activity was measured as previously described (81). Briefly, bacterial growth was measured by OD_600_, and cells were harvested by centrifugation and resuspended in potassium phosphate buffer (50 mM, pH 7.0). Cells were diluted 1:10 in Z buffer (60 mM Na_2_HPO_4_, 35 mM NaH_2_PO_4_, 1 mM KCl, 100 mM MgSO_4_, and 50 mM β-mercaptoethanol) and lysed using chloroform and 0.1% SDS. The enzyme reaction was started using *o*-nitrophenyl-β-d-galactopyranoside (ONPG, 4 mg/ml) dissolved in potassium phosphate buffer and stopped using sodium carbonate (1 M). The quenched reaction was centrifuged to remove cell debris, and the supernatant absorbance was read at *A*_420._ β-Galactosidase activity was calculated into Miller units (MU) using the following equation: MU = (1,000 × *A*_420_)/(time [min] × culture volume [ml] × OD_600_).

### Real-time PCR.

Five cultures of PA14 were grown as described above in metal-replete CDM, Fe-depleted, Mn-depleted, Zn-depleted, and metal-depleted CDM, and in metal-replete CDM in the presence of CP. Real-time PCR (RT-PCR) was performed as previously described (21). Briefly, cell pellets were stored in RNA*later* at –80°C. RNA was extracted using a Qiagen RNeasy kit, cDNA was synthesized, and RT-PCR was performed using TaqMan reagents (Roche) and a StepOnePlus system (Thermo Fisher). Relative expression was determined using the ΔΔ*C_T_* method. Expression was normalized to the 16S ribosomal gene. Primers and probes used are listed in Table S5.

### Azocasein protease activity assay.

Five biological replicates of PA14 were grown as described above. After 8 h of growth, cultures were centrifuged at 16,000 × *g* for 10 min. The supernatants were sterile filtered using 0.2-μm pore size filters (Costar) and stored at 4°C overnight. Cell pellets were lysed using BugBuster (Novagen) according to instructions. LasB activity was quantified as previously described (82). Briefly, 20 μl of supernatant was added to 0.5 ml of the 0.3% azocasein solution (50 mM Tris-HCl [pH 7.2] and 0.5 mM CaCl_2_ buffer supplemented with or without 10 μM ZnCl_2_) and incubated for 30 min at 37°C. The reaction was stopped with 0.5 ml of 10% trichloroacetic acid. The quenched reaction mixture was centrifuged at 16,000 × *g* for 20 min, and the supernatant absorbance was read at 400 nm using a Biotek Synergy HT plate reader. One unit of enzyme activity changes the *A*_400_ by 0.01. The enzyme activity was normalized to OD_600_.

### Minimal bactericidal concentration assay.

Three biological replicates of PA14 were grown as described above in metal-replete CDM, metal-depleted, and low-Mg CDM, and in metal-replete CDM in the presence of 10 μM CP. After 8 h of growth, the cultures were centrifuged, and the cells were resuspended in phosphate-buffered saline (PBS). The OD_600_ was measured, and PBS was inoculated to an OD_600_ of 0.05, which was subsequently diluted 1:10 into metal-replete CDM with no CaCl_2_ supplementation. The 1:10 dilution was used to inoculate 1 × 10^5^ CFU into 2 ml of metal-replete CDM with no CaCl_2_ supplementation containing 0, 1, 2, 4, 8, 16, 32, or 64 mg/liter polymyxin B. Serial dilutions of the inoculum were performed and plated on Pseudomonas isolation agar (PIA) and incubated overnight to ensure inoculum was at 1 × 10^5^ CFU/ml. Cultures were incubated for 18 h at 37°C. After growth, 10 μl of culture was spotted onto PIA, and the plates were incubated overnight at 37°C.

### Data availability.

The mass spectrometry proteomics data have been deposited to the ProteomeXchange Consortium via the PRIDE (83) partner repository with the dataset identifier PXD027638.
