# Supplementary material for: The Human Innate Immune Protein Calprotectin Elicits a Multimetal Starvation Response in Pseudomonas aeruginosa
Source: Microbiol Spectr. 2021 Sep 22;9(2):e00519-21. doi: 10.1128/Spectrum.00519-21 (PMC8557868; doi:10.1128/Spectrum.00519-21)
Supplement: SUPPLEMENTAL FILE 3 — Supplemental material. Download Spectrum.00519-21-s0003.pdf, PDF file, 0.5 MB [file spectrum.00519-21-s0003.pdf]

Supplementary Information for

The human innate immune protein calprotectin elicits a multi-metal starvation response in *Pseudomonas aeruginosa*

Cassandra E. Nelson[a*], Weilang Huang[a*], Emily M. Zygiel[b], Elizabeth M. Nolan[b#], Maureen A. Kane[a#], Amanda G. Oglesby[ac#]

[a]Department of Pharmaceutical Sciences, School of Pharmacy, University of Maryland, Baltimore, Baltimore, Maryland, USA

[b]Department of Chemistry, Massachusetts Institute of Technology, Cambridge, MA, USA

[c]Department of Microbiology and Immunology, School of Medicine, University of Maryland, Baltimore, Baltimore, Maryland, USA

*Contributed equally to this work

[#]Corresponding authors: Amanda Oglesby (aoglesby@rx.umaryland.edu), Elizabeth Nolan (lnolan@mit.edu), and Maureen Kane (mkane@rx.umaryland.edu)

Table S1: *Excel file of proteins significantly changed by CP, Fe-depletion, Zn-depletion, Mn-depletion, and metal-depletion*

Table S2 Network analysis of significantly changed proteins

Condition	Number of nodes[1]	Number of edges[2]	Expected number of edges[3]	PPI Enrichment p-value
Upregulated by CP treatment	93	328	57	< 1.0x10^{-16}
Downregulated by CP treatment	72	86	42	< 1.21x10^{-9}
Upregulated by Mn depletion	29	1	3	0.93
Downregulated by Mn depletion	17	2	3	0.762
Upregulated by Mn depletion, 0.5 LFC	72* (1)	27	27	0.508
Downregulated by Mn depletion, 0.5 LFC	50* (2)	11	16	0.908
Upregulated by Fe depletion	95* (1)	370	57	< 1.0x10^{-16}
Downregulated by Fe depletion	122* (1)	279	134	< 1.0x10^{-16}
Upregulated by Zn depletion	76* (1)	83	26	< 1.0x10^{-16}
Downregulated by Zn depletion	43* (2)	16	13	0.249
Upregulated by metal depletion	112	450	77	< 1.0x10^{-16}
Downregulated by metal depletion	190* (8)	430	237	< 1.0x10^{-16}

[1]Nodes are the proteins
[2]Edges are the interactions, based on known interactions from curated databases and experimental data, and predicted interactions from gene neighborhoods, gene fusions, and gene co-occurrence, from textmining, co-expression, and protein homology
[3]Expected edges are the number of interactions expected from a similar sample size of random genes from the genome
[4]PPI Enrichment p-value is the confidence the number of edges is due to biological connection versus random chance
*PAO1 proteins, some conditions have proteins that are unique to PA14. STRING database version 10.5 only has PAO1 in database. Numbers in parentheses are the number of proteins unique to PA14

Table S3: *Excel file of the comparisons between CP and metal depletion regulons*

Table S4 MBC of Polymyxin B after preculture with CP

Condition	MBC Range (mg/L)	Geometric Mean (mg/L)
Metal-replete	8-32	16
Metal-depleted	64-128	102
CP Treatment	32-128	64
Low Mg	32-64	51

Table S5 Components of metal-depleted chemically-defined medium (CDM)

**Salt**	**Final Concentration (mM)**
$(NH_4)_2SO_4$	51.8
KH_2PO_4	9.85
NaH_2PO_4	40.2
$CaCl_2$	0.0337
$NaCl_2$	0.0992
$MgSO_4$	2.05
**Amino Acid**	**Final Concentration (mM)**
L-Cysteine	1.98
L-Aspartic acid	18.0
L-Glutamic acid	16.3
L-Proline	20.8
L-Arginine	2.07
L-Glycine	32.0
L-Histidine	3.09
L-Lysine HCl	3.29
L-Serine	22.8
L-Valine	4.10
L-Tyrosine	0.993
L-Threonine	20.1
L-Alanine	26.9
L-Isoleucine	4.47
L-Phenylalanine	4.47
L-Tryptophan	0.294
L-Methionine	1.21
**Other**	**Final Concentration (mM)**
D-Glucose	20.2
Thiamine HCl	0.00178
Nicotinic Acid	1.21

Table S6 Primers and probes used in this study

Name	Sequence (5' to 3')	Reference
16S Forward	GGTGGTTCAGCAAGTTGGATGTG	(1)
16S Reverse	CCAGGTGGTCGCCTTCGC	(1)
znuA Forward	GGCTCGACGGGAAACTC	(2)
znuA Reverse	CGTAGGCCTCCTCGAAATAG	(2)
znuA probe	CG GCA AGC CTT TCT TCG TCT	(2)
zur Forward	CGT CGGCTGCAACAA	This study
zur Reverse	ATGGCCCGGCTGATA	This study
zur Probe	CACCAGGGCCAGTTCCTCATCTG	This study
lip2 Forward	GTCAATCCCGACCTCAA	(3)
lip2 Reverse	GTTCGTAGAGGCTGAAGAA	(3)
lip2 Probe	AGTGCGGCTGTTCGAGCTGAAA	(3)
hsiB2 Forward	TGCCGTTGAAGCTACTG	(3)
hsiB2 Reverse	CGTCGAAGGTCATCTTGT	(3)
hsiB2 Probe	CAAGGTGGAGGACCGCAAGCC	(3)
clpV2 Forward		This study
clipV2 Reverse		This study
clipV2 Probe		This study
xcpP Forward	CGC GGA CGA CAT TAC AA	This study
xcpP Reverse	TCT TCG GCG GGT TCT	This study
xcpP Probe	CGA TCG AAC AAC TGC AAA GCC TGC	This study
xcpT Forward	CCA AGG GCG ACA TCA AG	This study
xcpT Reverse	GGT AGC CGT CCT TGT TC	This study
xcpT Probe	AAG CTG GAC AAC TTC GCC TAT CCG	This study

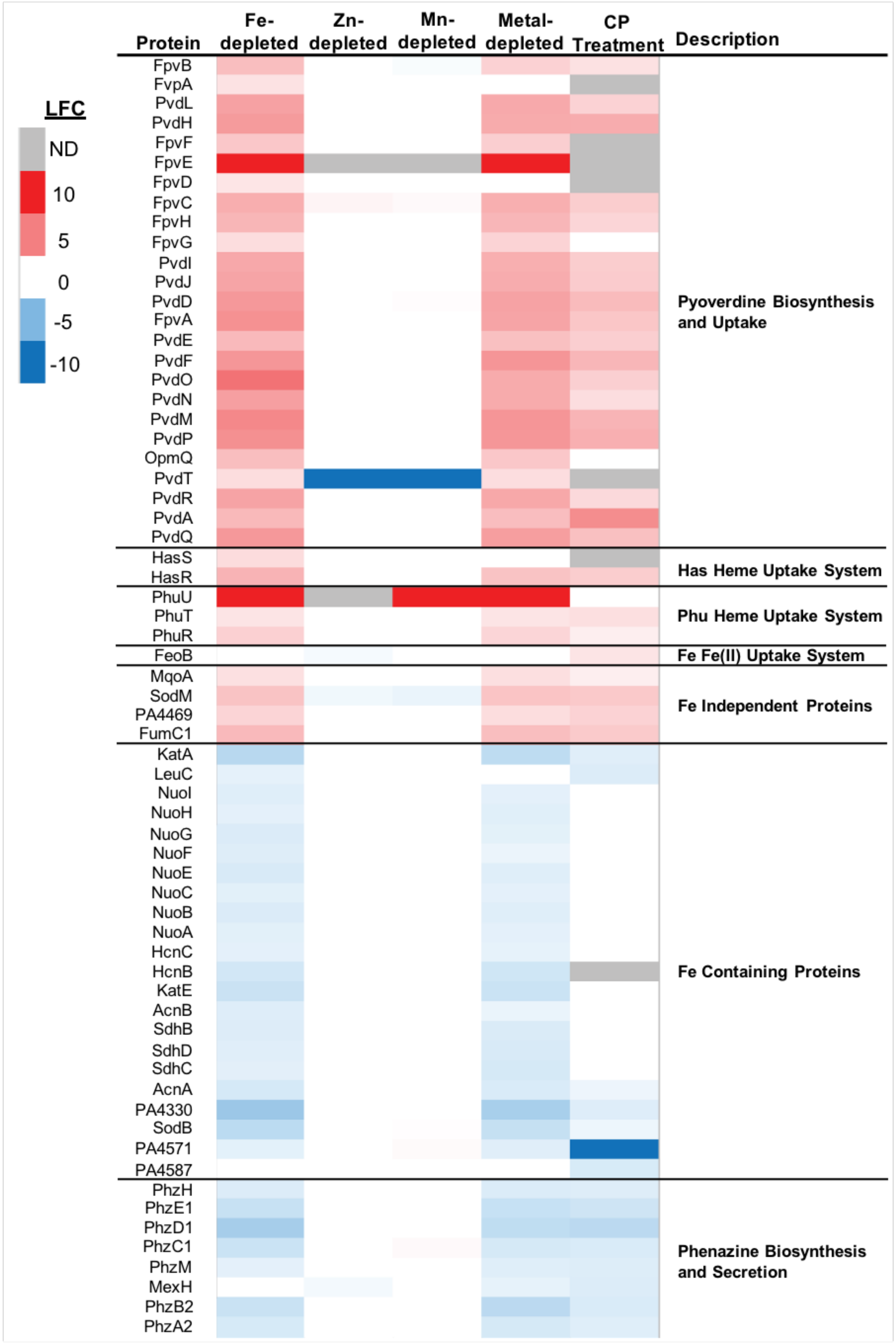
LFC
ND
10
5
0
-5
-10
Protein
Fe-depleted
Zn-depleted
Mn-depleted
Metal-depleted
CP Treatment
Description
FpvB
FvpA
PvdL
PvdH
FpvF
FpvE
FpvD
FpvC
FpvH
FpvG
PvdI
PvdJ
PvdD
FpvA
PvdE
PvdF
PvdO
PvdN
PvdM
PvdP
OpmQ
PvdT
PvdR
PvdA
PvdQ
Pyoverdine Biosynthesis and Uptake
HasS
HasR
Has Heme Uptake System
PhuU
PhuT
PhuR
Phu Heme Uptake System
FeoB
Fe Fe(II) Uptake System
MqoA
SodM
PA4469
FumC1
Fe Independent Proteins
KatA
LeuC
NuoI
NuoH
NuoG
NuoF
NuoE
NuoC
NuoB
NuoA
HcnC
HcnB
KatE
AcnB
SdhB
SdhD
SdhC
AcnA
PA4330
SodB
PA4571
PA4587
Fe Containing Proteins
PhzH
PhzE1
PhzD1
PhzC1
PhzM
MexH
PhzB2
PhzA2
Phenazine Biosynthesis and Secretion

Figure S1. Heatmap of Fe-regulated proteins. The $\log_2$-fold change (LFC) is shown for all significantly ($p<0.05$) changed proteins. Protein expression was compared to the metal-replete CDM control. n=5

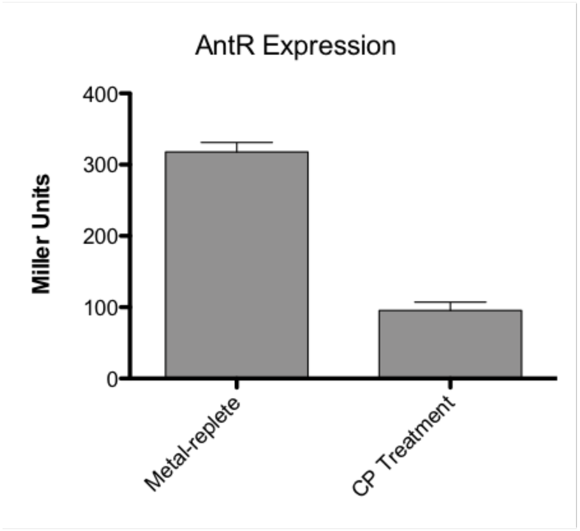

Figure S2. AntR expression is repressed by CP in both CDM and Tris:TSB. PA14/P*antR*-‘*lacZ*-SD was grown in meal-replete CDM in the presence or absence of 10 μM CP. AntR expression was determined by measuring β-galatosidase activity. Significance was determined by Students Two-Tailed T-Test, *$p<1.6x10^{-6}$, n=5.

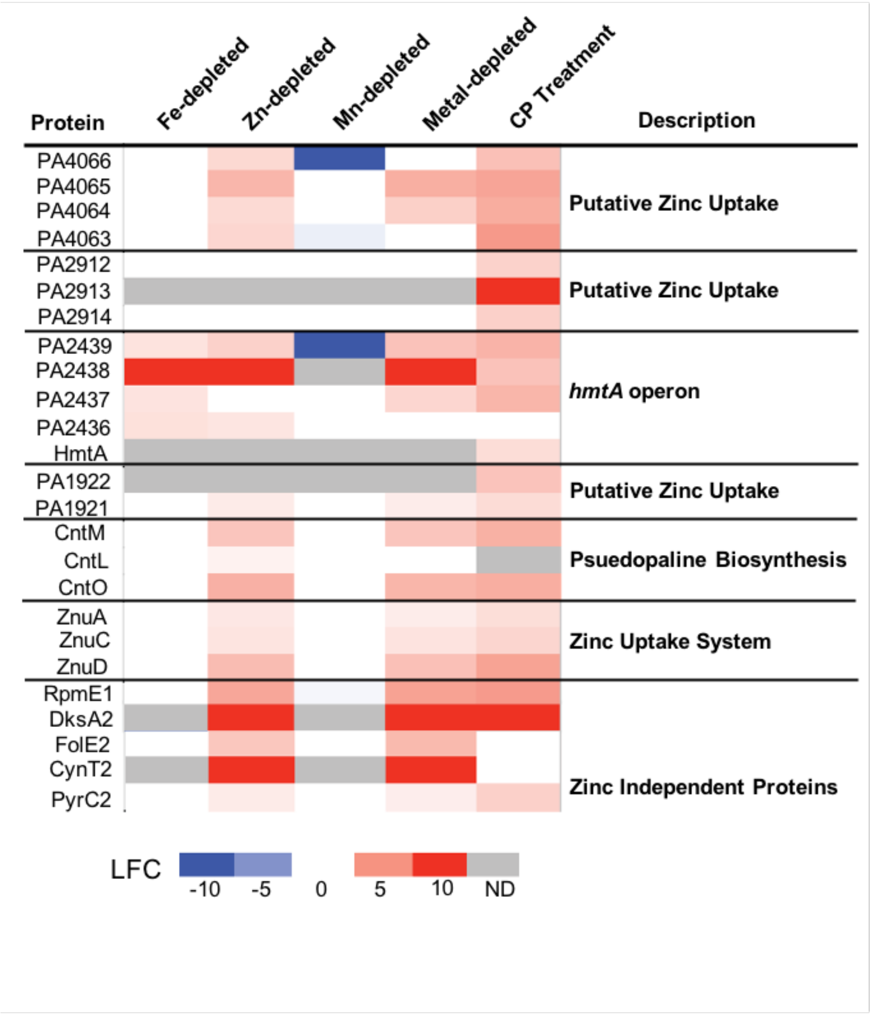

Figure S3. Heatmap of the Zn-starvation response. The protein expression was compared to the metal-replete CDM control. The $\log_2$-fold change (LFC) is shown for all significantly ($p<0.05$) changed proteins. n=5

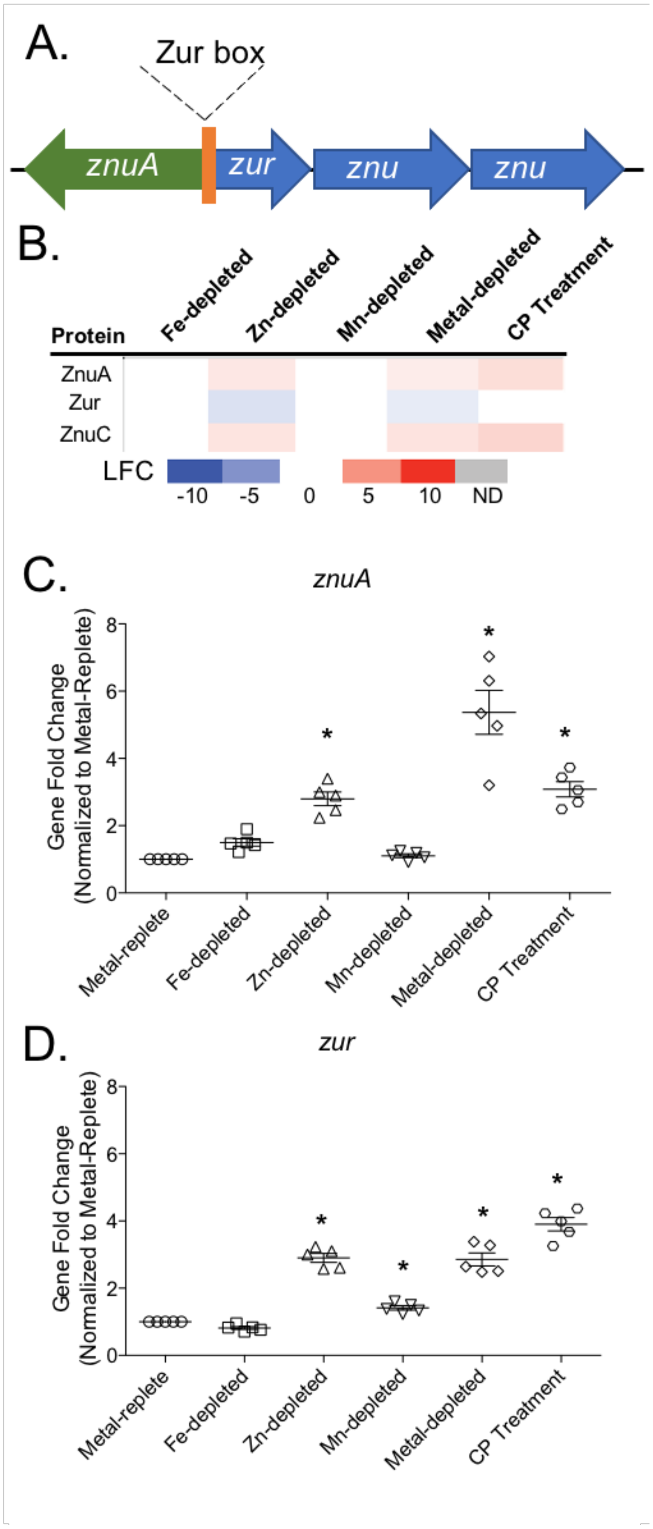

Figure S4. Zur is post-transcriptionally repressed during Fe starvation. A. Genetic organization of *znuA* and the *zur-znuCB* operon. *znuA* is transcribed in the opposite direction of

the *zur-znuCB* operon and shares a 70-bp intergenic region containing a Zur box. 1” = 1 mbp **B.** Protein expression of ZnuA, Zur, and ZnuC. The protein expression was compared to the metal-replete control. The $\log_2$-fold change (LFC) is shown for all significantly ($p<0.05$) changed proteins. Gene expression of *zur* (**C.**) and *znuA* (**D.**) was measured by RT-PCR and compared to the metal-replete CDM control. Significance was determined by one-way ANOVA with Dunnet’s multiple comparisons test. *$p<0.05$, n=5 for all experiments shown.

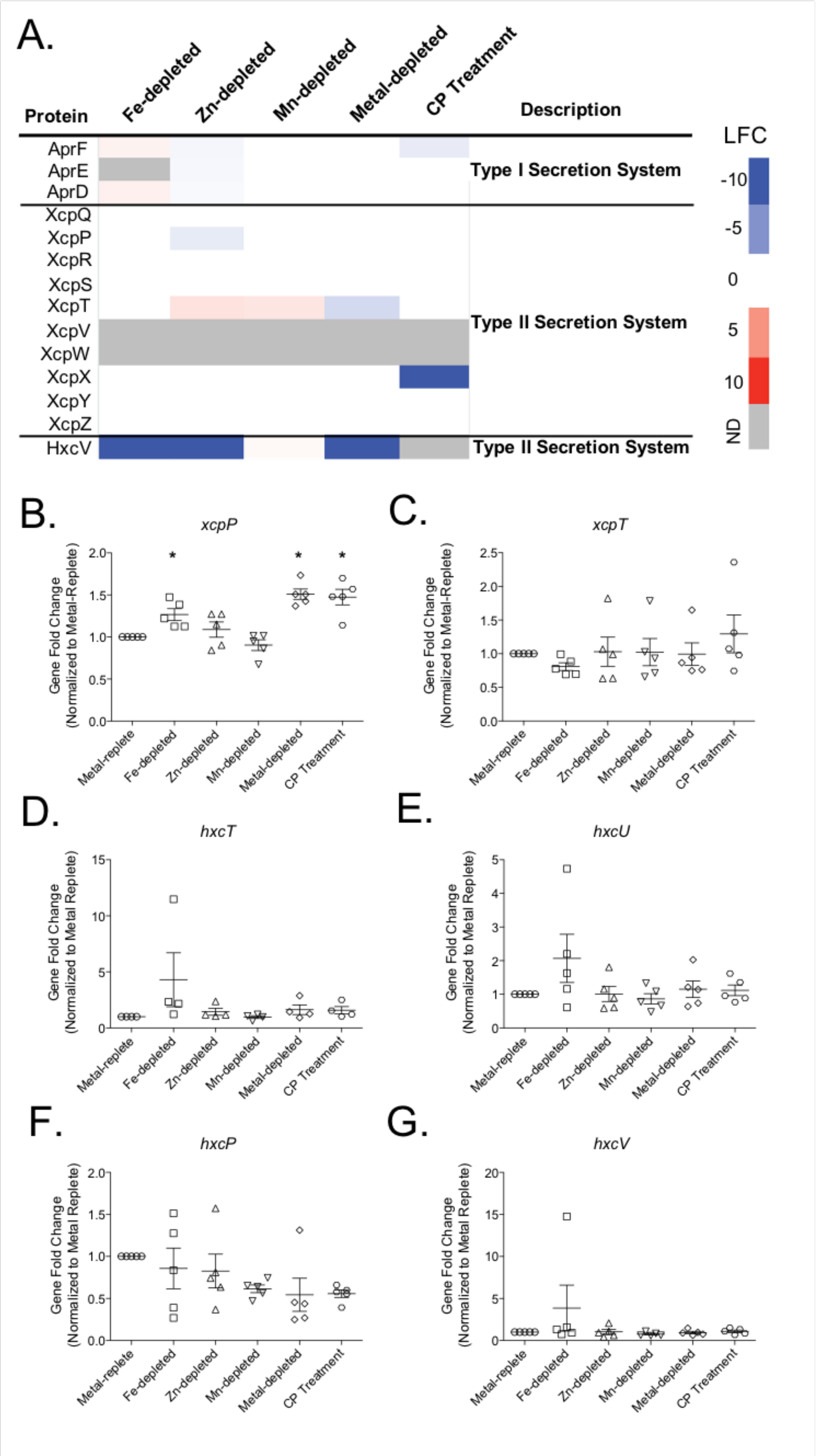
A.
Protein
Fe-depleted
Zn-depleted
Mn-depleted
Metal-depleted
CP Treatment
Description
AprF
AprE
AprD
Type I Secretion System
XcpQ
XcpP
XcpR
XcpS
XcpT
XcpV
XcpW
XcpX
XcpY
XcpZ
Type II Secretion System
HxcV
Type II Secretion System
LFC
-10
-5
0
5
10
ND
B.
xcpP
Gene Fold Change (Normalized to Metal-Replete)
Metal-replete
C.
xcpT
D.
hxcT
Gene Fold Change (Normalized to Metal Replete)
E.
hxcU
F.
hxcP
G.
hxcV

Figure S5. T2SS and T1SS expression is not strongly regulated by Fe, Zn, or Mn. A. Protein expression of the Apr, Xcp, and Hxc secretion systems. The protein expression was compared to the metal-replete CDM condition. The log_2-fold change (LFC) is shown for all significantly ($p<0.05$) changed proteins. Gene expression of *xcpP* (**B.**), *xcpT* (**C.**), *hxcT* (**D.**), *hxcU* (**E.**), *hxcP* (**F.**) and *hxcV* (**G.)** was measured by RT-PCR and expression was compared to the metal-replete CDM control. Significance was determined using a one-way ANOVA with Dunnett's multiple comparison. *$p<0.05$, $n=5$

Quantitative label-free proteomics. Two independent proteomics experiments were performed each with five biological replicates. The media for both experiments was prepared as described above as a single batch, inoculated with the same five overnight cultures, and samples were collected for both experiments at the same time to limit variability. For the first experiment, PA14 was grown in CDM with and without 10 μM CP. For the second experiment, PA14 was grown in CDM, Fe-depleted, Mn-depleted, Zn-depleted, metal-depleted CDM. Quantitative label-free proteomics was performed similar as previously described with modification(3-5). Cells were harvested by centrifugation and washed in phosphate-buffered saline prior to lysis in 4% sodium deoxycholate. Lysates were washed, reduced, alkylated, and trypsinolyzed on a filter. Tryptic peptides were separated using a nanoACQUITY UPLC analytical column (BEH130 C18, 1.7 µm, 75 µm x 200 mm; Waters) over a 165-min linear acetonitrile gradient (3 to 40%) with 0.1% formic acid on a Waters nanoAcquity UPLC system and analyzed on a coupled Thermo Scientific Orbitrap Fusion Lumos Tribrid mass spectrometer. Full scans were acquired at a resolution of 240,000, and precursors were selected for fragmentation by collision-induced dissociation (normalized collision energy at 35%) for a maximum 3 second cycle. Tandem mass spectra were searched against reference protein sequences of the *Pseudomonas aeruginosa* PA14 genome database (6) using the Sequest-HT and MS Amanda algorithms with a maximum precursor mass error tolerance of 10ppm (7, 8). Carbamidomethylation of cysteine and deamidation of asparagine and glutamine were treated as static and dynamic modifications, respectively. Resulting hits were validated at a maximum false-discovery rate (FDR) of 0.01 using a semi-supervised machine learning algorithm Percolator (9) Label-free quantifications were performed using Minora, an aligned accurate mass and retention time (AMRT) cluster quantification algorithm (Thermo Scientific). Protein abundance ratios between the CP-treated or metal-depleted cultures and the metal-replete cultures were measured by comparing the MS1 peak volumes of peptide ions, whose identities were confirmed by MS2 sequencing as described above. The thresholds for inclusion were as follows: significance ($p<0.05$) and expression (1 $\log_2$ fold change, LFC) equivalent to a 2-fold change. Gene function and pathway analysis was conducted using information from the *Pseudomonas* genome database (6) and the *Pseudomonas* metabolome database (10), and the STRING database (11).

Supplementary References

1. Cenciarini C, Courtois S, Raoult D, La Scola B. 2008. Influence of long time storage in mineral water on RNA stability of Pseudomonas aeruginosa and Escherichia coli after heat inactivation. PLoS One 3:e3443.
2. Nelson CE, Huang W, Brewer LK, Nguyen AT, Kane MA, Wilks A, Oglesby-Sherrouse AG. 2019. Proteomic Analysis of the Pseudomonas aeruginosa Iron Starvation Response Reveals PrrF Small Regulatory RNA-Dependent Iron Regulation of Twitching Motility, Amino Acid Metabolism, and Zinc Homeostasis Proteins. J Bacteriol 201.
3. Brewer LK, Huang W, Hackert BJ, Kane MA, Oglesby AG. 2020. Static Growth Promotes PrrF and 2-Alkyl-4(1H)-Quinolone Regulation of Type VI Secretion Protein Expression in Pseudomonas aeruginosa. J Bacteriol 202.
4. Defnet AE, Huang W, Polischak S, Yadav SK, Kane MA, Shapiro P, Deshpande DA. 2019. Effects of ATP-competitive and function-selective ERK inhibitors on airway smooth muscle cell proliferation. FASEB J 33:10833-10843.
5. Kim D, Chen R, Sheu M, Kim N, Kim S, Islam N, Wier EM, Wang G, Li A, Park A, Son W, Evans B, Yu V, Prizmic VP, Oh E, Wang Z, Yu J, Huang W, Archer NK, Hu Z, Clemetson N, Nelson AM, Chien A, Okoye GA, Miller LS, Ghiaur G, Kang S, Jones JW, Kane MA, Garza LA. 2019. Noncoding dsRNA induces retinoic acid synthesis to stimulate hair follicle regeneration via TLR3. Nat Commun 10:2811.
6. Winsor GL, Griffiths EJ, Lo R, Dhillon BK, Shay JA, Brinkman FS. 2016. Enhanced annotations and features for comparing thousands of Pseudomonas genomes in the Pseudomonas genome database. Nucleic Acids Res 44:D646-53.
7. Eng JK, Fischer B, Grossmann J, Maccoss MJ. 2008. A fast SEQUEST cross correlation algorithm. J Proteome Res 7:4598-602.
8. Dorfer V, Pichler P, Stranzl T, Stadlmann J, Taus T, Winkler S, Mechtler K. 2014. MS Amanda, a universal identification algorithm optimized for high accuracy tandem mass spectra. J Proteome Res 13:3679-84.
9. Käll L, Canterbury JD, Weston J, Noble WS, MacCoss MJ. 2007. Semi-supervised learning for peptide identification from shotgun proteomics datasets. Nat Methods 4:923-5.
10. Huang W, Brewer LK, Jones JW, Nguyen AT, Marcu A, Wishart DS, Oglesby-Sherrouse AG, Kane MA, Wilks A. 2018. PAMDB: a comprehensive Pseudomonas aeruginosa metabolome database. Nucleic Acids Res 46:D575-d580.
11. Szklarczyk D, Morris JH, Cook H, Kuhn M, Wyder S, Simonovic M, Santos A, Doncheva NT, Roth A, Bork P, Jensen LJ, von Mering C. 2017. The STRING database in 2017: quality-controlled protein-protein association networks, made broadly accessible. Nucleic Acids Res 45:D362-d368.
